# Supplementary material for: The PML1-WDR5 axis regulates H3K4me3 marks and promotes stemness of estrogen receptor-positive breast cancer
Source: Res Sq. 2023 Sep 8:rs.3.rs-3266720. Preprint. [Version 1] doi: 10.21203/rs.3.rs-3266720/v1 (PMC10503857; doi:10.21203/rs.3.rs-3266720/v1)
Supplement: Supplement 1 [file NIHPPRS3266720V1-supplement-1.pdf]

Supplementary Files

This is a list of supplementary files associated with this preprint. Click to download.

- SupplementaryFigures.pdf
- SupplementaryMethodsandFigureLegends.docx
